# Notices

**Published:** 1871-04

**Authors:** 


### *American Medical Association,*

Office of Permanent Secretary, Wm. B. Atkinson, M.D., 1400 Pine street, S. W. cor. Broad, Philadelphia.

The twenty-second annual session will be held in San Francisco, Cal., May 2, 1871, at 11 A. M.

The following Committees are expected to report:—

On Cultivation of the Cinchona Tree—Dr. Lemuel J. Deal, Pennsylvania, Chairman.

On Inebriate Asylums—Dr. C. H. Nichols, D.C., Chairman.

On Institutions for Inebriates—Dr. Joseph Parrish, Pennsylvania, Chairman.

On the Structure of the White Blood Corpuscles—Dr. J. G. Richardson, Pa., Chairman.

On Vaccination—Dr. Henry A. Martin, Mass., Chairman.

On the Comparative Merits of Syme's and Pirogoff's Operations—Dr. Geo. A. Otis, U. S. A., Chairman.

On Lithotrity—Dr. E. M. Moore, New York, Chairman.

On Veterinary Medicine—Dr. Samuel D. Gross, Pa., Chairman.

On Protest of Naval Surgeons, etc.—Dr. W. S. W. Ruschenberger, U. S. N., Chairman.

On National Medical School—Dr. Francis Gurney Smith, Pa., Chairman.

On American Medical Association Journal—Dr. James P. White, New York, Chairman.

On Criminal Abortion—Dr. D. A. O'Donnell, Maryland, Chairman.

On Nomenclature of Diseases—Dr. Francis Gurney Smith, Pa., Chairman.

On National System of Quarantine—Dr. J. C. Tucker, California, Chairman.

On what, if any, Legislative means are expedient and advisable, to prevent the spread of Contagious Diseases—Dr. M. H. Henry, New York, Ch'n.

On Renewal of Prescriptions by Apothecaries without Authority—Dr. R. J. O'Sullivan, New York, Chairman.

On American Medical Necrology—Dr. C. C. Cox, D. C., Chairman.

On Medical Education—Dr. Ely Geddings, South Carolina, Chairman.

On Medical Literature—Dr. P. G. Robinson, Missouri, Chairman.

On Prize Essays—Dr. T. M. Logan, California, Chairman.

On the Climatology and Epidemics of—Maine, Dr. J. C. Weston; New Hampshire, Dr. P. A. Stackpole; Massachusetts, Dr. H. I. Bowditch; Rhode Island, Dr. C. W. Parsons; Connecticut, Dr. J. C. Jackson; New York, Dr. W. F. Thoms; New Jersey, Dr. C. F. J. Lehlbach; Pennsylvania, Dr. D. F. Condie; Maryland, Dr. C. H. Ohr; Georgia, Dr. Juriah Harriss; Missouri, Dr. F. E. Baumgarten; Alabama, Dr. R. F. Michel; Texas, Dr. S. M. Welsh; Illinois, Dr. R. C. Hamil; Indiana, Dr. J. F. Hibberd; District of Columbia, Dr. T. Antisell; Iowa, Dr. J. C. Hughes; Michigan, Dr. G. P. Andrews; Ohio, Dr. T. L. Neal; California, Dr. F. W. Hatch; Tennessee, Dr. B. W. Avent; West Virginia, Dr. E. A. Hildreth; Minnesota, Dr. Chas. N. Hewitt; Virginia, Dr. W. O. Owen; Delaware, Dr. L. B. Bush; Arkansas, Dr. G. W. Lawrence; Mississippi, Dr. J. P. Moore; Louisiana, Dr. S. M. Bemiss; Wisconsin, Dr. J. K. Bartlett; Kentucky, Dr. L. P. Yandell, Sr.; Oregon, Dr. E. R. Fisk; North Carolina, Dr. W. H. McKee.

Secretaries of all medical organizations are requested to forward lists of their delegates, as soon as elected, to the Permanent Secretary.

Any respectable physician who may desire to attend, but cannot do so as a delegate, may be made a *member by invitation*, upon the recommendation of the Committee of Arrangements.

W. B. Atkinson.

### *American Medical Association.*

Arrangements have been completed with the Northwestern and Union Pacific Railroads to carry members of the profession, who desire to attend the annual meeting of the American Medical Association on the 2d day of May, 1871, in San Francisco, from Chicago to Omaha and back for $24, and from Omaha to San Francisco and back for $125, making $149 for the round trip from this place.

Tickets can be purchased at any time after the 15th of April, and will be good for 60 days from date of purchase.

Physicians can take members of their own families with them at the same rates. Tickets from Chicago to Omaha and back can be purchased at the Ticket Office, 125 Lake street, *only*. For Omaha to San Francisco and back, at the office in Omaha. But all applicants for commutation tickets will be required to show certificates that they are to be recognized as actual members of the San Francisco meeting. Consequently, all in the Northwest who intend to go, had better send to me, without delay, their names and address, with the names of such members of their families as they desire to take with them.

N. S. Davis,

Sec. Committee on Railroads, 797 Wabash av., Chicago.

